# Cytosporone B Affects ATP Production of *Trypanosoma cruzi*: A Lethal Action Investigation

**DOI:** 10.1021/acsomega.5c09059

**Published:** 2025-12-10

**Authors:** Augusto Leonardo dos Santos, Maiara Amaral, Erica V. de Castro Levatti, Maiara Romanelli, Marcelo J. Pena Ferreira, Andre G. Tempone, Patrícia Sartorelli

**Affiliations:** † Instituto de Ciências Ambientais, Químicas e Farmacêuticas, 28105 Universidade Federal de São Paulo (Unifesp), Campus Diadema, Diadema, São Paulo 09972-270, Brazil; ‡ Pathophysiology Laboratory, 196591 Instituto Butantan, São Paulo, São Paulo 05503-900, Brazil; § Botany Department, Institute of Biosciences, University of São Paulo, São Paulo 05508-090, Brazil

## Abstract

Chagas disease is a neglected parasitic infection spreading worldwide and affecting 8–10 million people, with 14,000 deaths. The treatment is limited to two toxicity nitroheterocycle drugs, approved more than 50 years ago. Considering the urgent need for new therapies, secondary metabolites isolated from endophytic fungi may represent a useful source of bioactive molecules with antiparasitic activity, as described in the literature. In this study, we investigated the anti-*Trypanosoma cruzi* activity of seven compounds isolated from endophytic fungi present in leaves of the plant *Casearia arborea*. The compounds dothiorelones A (1), B (**2**), P (**3**), and Q (**4**), cytosporones A (**5**) and B (**6**) were isolated from *Phomopsis* sp. CarGL23, and cytochalasin H (**7**), from *Diaporthe* sp. CarGL8. Compound **6** (Csn-B) was designated the most selective agent of the panel, exhibiting potent activity against both relevant stages of *T. cruzi*. Specifically, it achieved IC_50_ values of 36.5 μM against trypomastigotes and 9.1 μM against intracellular amastigotes. Given its superior selectivity index, a key criterion for drug discovery progression, Csn-B was prioritized for mechanistic investigation. Subsequent studies focused on understanding its lethal action profile using advanced spectrofluorimetric and flow cytometry assays. Using JC-1 as a fluorescent probe, our data revealed that Csn-B caused the depolarization of the mitochondrial membrane potential, without affecting the plasma membrane permeability of the trypomastigotes, as observed by the SYTOX Green dye. Targeting the bioenergetic system, Csn-B resulted in a decrease in the ATP levels of the parasite, affecting the viability of the cell. This study revealed a promising and selective anti-*T. cruzi* profile of these natural compounds and suggests Csn-B as a hit compound for future investigations for Chagas disease.

## Graphical abstract

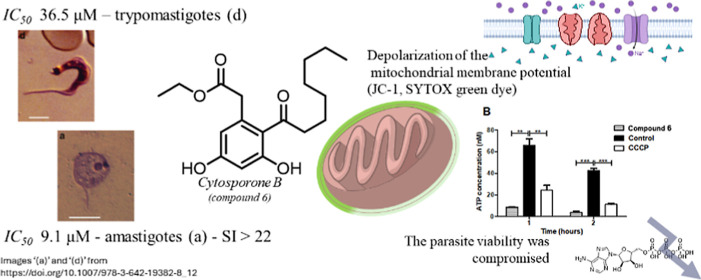

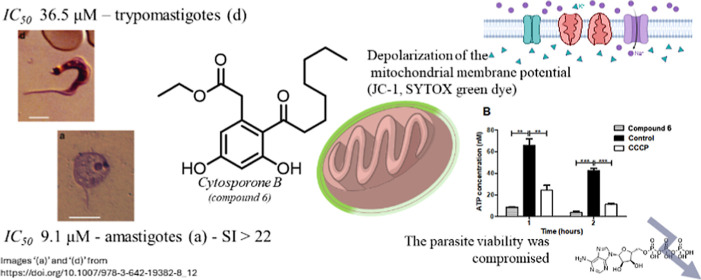

## Introduction

Chagas disease is a Neglected Tropical Disease caused by the protozoan parasite *Trypanosoma cruzi* *.* It is endemic in 21 countries of Latin America and affects 7 million people worldwide, with 14,000 deaths annually. Around 30–40% of patients can develop complications as cardiomyopathy, megaviscera, megaesophagus, or megacolon, leading to the patient’s death.^1^ The treatment is limited to two toxic nitroheterocycle drugs (nifurtimox and benznidazole), with limited efficacy during the chronic phase of the disease.^2^ These facts, associated with parasite resistance, demonstrate the urgent need for new chemotherapy candidates to treat Chagas disease.^3^

Considering that approximately 50% of all approved drugs were inspired by natural scaffolds, natural products can be a promising source of bioactive molecules with a wide variety of pharmacological activities. Since 1981, the approval of antiparasitic drugs has been limited, with only 18 small molecules approved.^4^ Despite the substantial global burden of parasitic diseases, awareness remains low, particularly among economically privileged populations. In 1987, two natural compounds were approved (artemisinin and ivermectin), and seven natural-derived compounds were approved in the last 30 years, including fansimef in 1985, artemether and arinate in 1987, ornidyl in 1990, artemotil in 2000, etaquin and moxidectin in 2018. For Chagas disease, there have been some advances in recent years, but no success in the current clinical trial investigations of synthetic drugs and associations for trypanosomiasis. Chagas disease is a neglected disease with no drug effective for the chronic phase since the last century, as in the last 30 years, it has been very difficult to find natural alternatives with antiparasitic properties, especially for chagas disease, which remains challenging.^4,5^

Endophytes are a complex community of microorganisms that colonize the internal tissues of higher plants without causing symptoms for the host. This association may regard the production of bioactive metabolites that protect the host against herbivores and pathogenic microorganisms. Furthermore, secondary metabolites derived from microorganisms, remain an important source of structurally diverse and biologically active compounds and are often considered more sustainable and environmentally friendly compared to fully synthetic approaches, as their biosynthesis occurs under mild conditions using renewable resources.^6^ *Casearia arborea* (Salicaceae) was selected as the host plant due to the remarkable bioactive potential reported for species of this genus. Furthermore, *Casearia* plants are known to produce clerodane diterpenes and related metabolites with documented antiparasitic effects, including activity against *T. cruzi*.^7^ Thus, considering that *C. arborea* thrives in tropical environments rich in microbial diversity, its endophytic community may harbor unique biosynthetic pathways leading to metabolites with biological potential.^8,9^ In this work, we aimed to evaluate the anti-*T. cruzi* activity of six cytosporone derivatives (compounds **1**–**6**) and cytochalasin H (compound **7**) from endophytic fungi isolated from leaves of *C. arborea*. In addition, the mechanism of action of the most promising compound was studied by spectrofluorimetric and flow cytometry techniques.

## Material and Methods

### Plant Material

Leaves of *C. arborea* Rich. were collected in the Atlantic Forest, Alfenas city, MG, Brazil (coordinates S 21°22′53.8″ W 045°55′46.4″), in June 2016. João Pedro Costa Elias performed the botanical identification from Universidade Federal de Alfenas (UNIFAL-Alfenas city, MG, Brazil), and a voucher specimen (Elias J.P.C. 02) was deposited in the SPF herbarium, São Paulo University.

### Isolation and Cultivation of Endophytic Fungi

The methodology used for the isolation of endophytic fungi was based on the procedure described in the literature. Briefly, the methods for incubation and growth of endophytic fungi were described previously.^8^

### Dereplication of Secondary Metabolites

Compounds **1**–**7** (Suppl. A) were obtained as secondary metabolites of *Phomopsis* sp. and *Diarpothe* sp., both endophytic fungi found in *C. arborea* (Salicaceae). Dothiorelones A (**1**), B (**2**), P (**3**), and Q (**4**), cytosporones A (**5**) and B (**6**) were found in molecular networking dereplication of *Phomopsis* sp. CarGL23, then purified through HPLC-DAD, and characterization was performed using spectrometric and spectroscopic data.^8^ Cytochalasin H (compound **7**) was detected in the *Diaporthe* sp. CarGL8 metabolome from molecular networking according to a characteristic HRMS/MS fragmentation pattern of cytochalasin derivative, which led to the isolation of this compound by SiO_2_ and Sephadex LH-20 chromatography.^9^ All compounds were identified through 1D and 2D-NMR spectroscopy and HRMS/MS analysis^8,9^ (Suppl. B and C).

### Animals

All experimental procedures used in this work were previously approved by the Animal Care and Use Committee from Instituto Adolfo Lutz, Secretary of Health of Sao Paulo State (Project number CEUA 05/2018) in agreement with the Guide for the Care and Use of Laboratory Animals from the National Academy of Science.

### Parasites and Cells

Murine conjunctive cells (NCTC clone 929, ATCC) and rhesus monkey kidney cells (LLC-MK2, ATCC CCL 7) were maintained in RPMI-1640 supplemented with 10% fetal bovine serum (FBS) at 37 °C in 5% CO_2_ humidified incubator. Peritoneal macrophages were collected by washing the peritoneal cavity of BALB/c mice with RPMI-1640 medium (Sigma-Aldrich) supplemented with 10% FBS, and were maintained at 37 °C in a 5% CO_2_ humidified incubator. *T. cruzi* trypomastigotes (Y strain) were cultivated by continuous in vitro passage in LLC-MK2, in RPMI-1640 supplemented with 2% FBS at 37 °C in 5% CO_2_ humidified incubator. Antitrypomastigote activity was evaluated in vitro using trypomastigotes obtained from infected LLC-MK2 cell cultures. The motile trypomastigotes, spontaneously released into the supernatant, were collected by centrifugation (2100*g* for 10 min), washed three times with cold PBS to remove cell debris and culture medium, and finally resuspended in fresh RPMI-1640 medium.

### Cytotoxic Assay

Cytotoxicity was evaluated in vitro using fibroblast cells (NCTC clone 929) seeded in a 96-well plate (6 × 10^4^/well). The compounds were diluted in DMSO (30 mM) and diluted in RPMI-1640 medium without phenol red compounds using serial dilution from 1.56 to–200 μM. The compounds were incubated with the cells for 48 h at 37 °C in 5% CO_2_ humidified incubator. The MTT assay was used for viability determination.^10^ After the final incubation period, 20 μL of MTT (5 mg/mL) was added for 4 h and the plate was kept at 37 °C in 5% CO_2_ humidified incubator. To solubilize the formazan, 80 μL of SDS (10% v/v) was added and the microplate was incubated for 24 h at 25 °C. The microplate was read at 570 nm in a plate spectrophotometer (Varioskan, Thermo, USA).

### Antitrypomastigote Assay

Antitrypomastigote activity was evaluated in vitro using trypomastigotes obtained from infected LLC-MK2 cell cultures. Briefly, LLC-MK2 cells (1 × 10^6^ cells/well) were incubated with increasing concentrations of the test compounds (up to 150 μM) in RPMI-1640 medium supplemented with 10% fetal bovine serum, at 37 °C in a humidified atmosphere containing 5% CO_2_ for 24 h. After incubation, resazurin sodium salt (final concentration 44 μM) was added to each well, and the plates were further incubated for an additional 24 h under the same conditions. Resazurin is a redox-sensitive dye that is reduced by metabolically active parasites to the fluorescent compound resorufin, allowing the quantification of viable parasites.

The conversion of resazurin to resorufin was measured spectrophotometrically by reading the absorbance at 570 nm. The percentage of parasite viability was calculated relative to untreated controls. Benznidazole was used as a standard drug, and wells containing only medium served as negative controls.^11^

### Antiamastigote Assay

Macrophages were seeded in 16-well chamber slides (NUNC Thermo Fisher Scientific, USA) and after 24 h infected with trypomastigotes (1:5, host cell/parasite ratio) for 2 h. Subsequently, infected cells were incubated with increasing concentrations of the compounds (up to 60 μM) for 48 h at 37 °C in 5% CO_2_ humidified incubator. After treatment, the cells were fixed and stained with Giemsa, and the slides were analyzed under a digital light microscope (EVOS M500, Thermo Fisher Scientific, USA). The infection index was determined by counting 200 macrophages and calculating the percentage of infected cells multiplied by the average number of amastigotes per infected macrophage. Benznidazole was used as a standard reference drug.^12^

### Determination of the IC_50_ Value for Mechanism of Action Studies

To specifically determine the IC_50_ value for mechanism-of-action studies requiring a short exposure time, the standard protocol was modified to a 3 h compound incubation. Trypomastigotes, purified from infected LLC-MK2 cell cultures, were first resuspended in RPMI-1640 medium supplemented with 10% fetal bovine serum. Briefly, 1 × 10^6^ trypomastigotes per well were dispensed and immediately incubated with increasing concentrations of the test compound (up to 150 μM) in a humidified 5% CO_2_ incubator for a short period of 3 h. Following this exposure, 20 μL/well of MTT (3-(4,5-dimethylthiazol-2-yl)-2,5-diphenyltetrazolium bromide) was added (5 mg/mL solution), and the plates were further incubated for an additional 3 h under the same conditions.^13^ Viable parasite density was then quantified based on the metabolic reduction of MTT, which was measured spectrophotometrically by reading the absorbance at 570 nm. This short incubation period allows for the determination of the compound’s immediate potency before secondary effects might occur.

### Plasma Membrane Integrity

The plasma membrane permeability was investigated using the fluorescent prove SYTOX Grenn. SYTOX Green is a high-affinity nucleic acid stain that selectively penetrates cells with compromised plasma membranes, allowing the identification of dead or membrane-damaged cells by producing a bright green fluorescence upon binding to DNA. Trypomastigotes (2 × 10^6^/well) were collected and incubated in a 96-well black microplate with 1 μM SYTOX Green (Molecular Probes, EUA) in HANKS balanced salt solution (HBSS, Sigma-Aldrich) supplemented with 10 mM d-glucose (HBSS + Glu) for 15 min at 24 °C in the dark. After this period, Csn-B (**6**) was added (55 μM), and a kinetic curve of fluorescence intensity was set up every 20 min for 120 min using a microplate reader.^14^

### Mitochondrial Membrane Electric Potential (ΔΨ_m_)

Mitochondrial membrane potential (ΔΨ_m_) was evaluated using the JC-1 fluorescent probe, which accumulates in mitochondria in a potential-dependent manner, forming red-fluorescent aggregates in healthy cells with high ΔΨ_m_ and remaining as green-fluorescent monomers in cells with depolarized mitochondrial membranes. Trypomastigotes (2 × 10^6^/well) were incubated with Csn-B (**6**) (55 μM) for 1 and 2 h, at 37 °C in HBSS + Glu. Carbonyl cyanide *m*-chlorophenyl hydrazone (CCCP) 100 μM was used as a positive control. After this period, parasites were washed in HBSS + Glu and resuspended with JC-1 dye (10 μM) in the dark for 10 min at 37 °C. Parasites were analyzed by flow cytometry (Attune NxT Flow Cytometer; Thermo Fisher Scientific, USA).^14,15^

### ATP Levels

Intracellular ATP levels were measured using the ATP Determination Kit (Molecular Probes, Thermo Fisher Scientific, USA), based on the luciferin–luciferase bioluminescence reaction, in which the emitted light intensity is directly proportional to the ATP concentration present in the sample. Trypomastigotes (2 × 10^6^/well) were treated with Csn-B (**6**) (55 μM) for 1 and 2 h. Carbonyl cyanide *m*-chlorophenyl hydrazone (CCCP) 100 μM was used as a positive control. After treatment, trypomastigotes were lysed using HBSS + Glu with 0.5% Triton X-100 and mixed with a standard reaction buffer (ATP Determination kit, Molecular Probes, USA) containing DTT (1 mM), luciferin (0.5 mM), and firefly luciferase (1.25 μg/mL).^14,15^

### Statistical Analysis

Each experiment was performed in duplicate, and the assay was repeated at least twice. All statistical analyses were conducted using GraphPad Prism 5.0 software. Statistical methods were specifically applied based on the type of data being analyzed: **i**) Determination of CC_50_ and IC_50_ values (Table 1): The half-maximal inhibitory concentration (IC_50_) for antiparasitic activity and the CC_50_ for 50% cytotoxic concentration for host cells were determined using nonlinear regression to fit the dose–response data to a sigmoid curve (variable slope model). (ii) Comparison of Experimental Groups (Figures 2 and 3): To evaluate the statistical significance between different experimental treatment groups (as presented in Figures 2 and 3), the data were analyzed using one-way analysis of variance (ANOVA), followed by the Tukey Multiple Comparison Test to identify specific significant differences between all pairs of means. A difference was considered statistically significant when the calculated *p*-value was less than 0.05 (*p* < 0.05).

**Table 1:** Anti-*Trypanosoma cruzi* Activity and Cytotoxicity of the Compounds **1–7** ^a^

compound	IC_50_ (μM) ± SD		CC_50_ (μM) ± SD	SI
	Trypomastigote	Amastigote		
**1**	NA	NA	>200	ND
**2**	NA	NA	>200	ND
**3**	NA	NA	>200	ND
**4**	NA	43.0 ± 9.0	>200	>4.6
**5**	NA	29.9 ± 0.5	>200	>6.7
**6**	36.5 ± 1.9	9.1 ± 3.2	>200	>22.0
**7**	47.9 ± 6.3	NA	>200	ND
Benznidazole	17.7 ± 1.9	5.0 ± 1.5	190.6 ± 13.4	38.1

a IC_50_, 50% inhibitory concentration; cc_50_, 50% cytotoxicity concentration; Supporting Information, selectivity index, given by the ratio between CC_50_ in NCTC cells and IC_50_ in intracellular amastigotes; SD, standard deviation. dothiorelones A (**1**), B (**2**), P (**3**) and Q (**4**), cytosporones A (**5**) and B (**6**), cytochalasin H (**7**).

**Figure 1:**
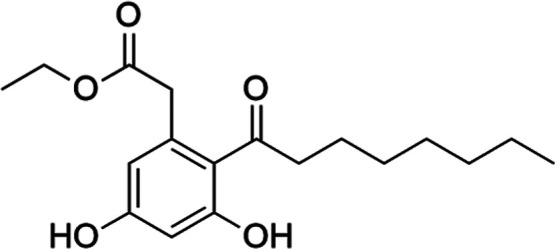
Chemical structure of compound **6**, Cytosporone B (Csn-B).

**Figure 2:**
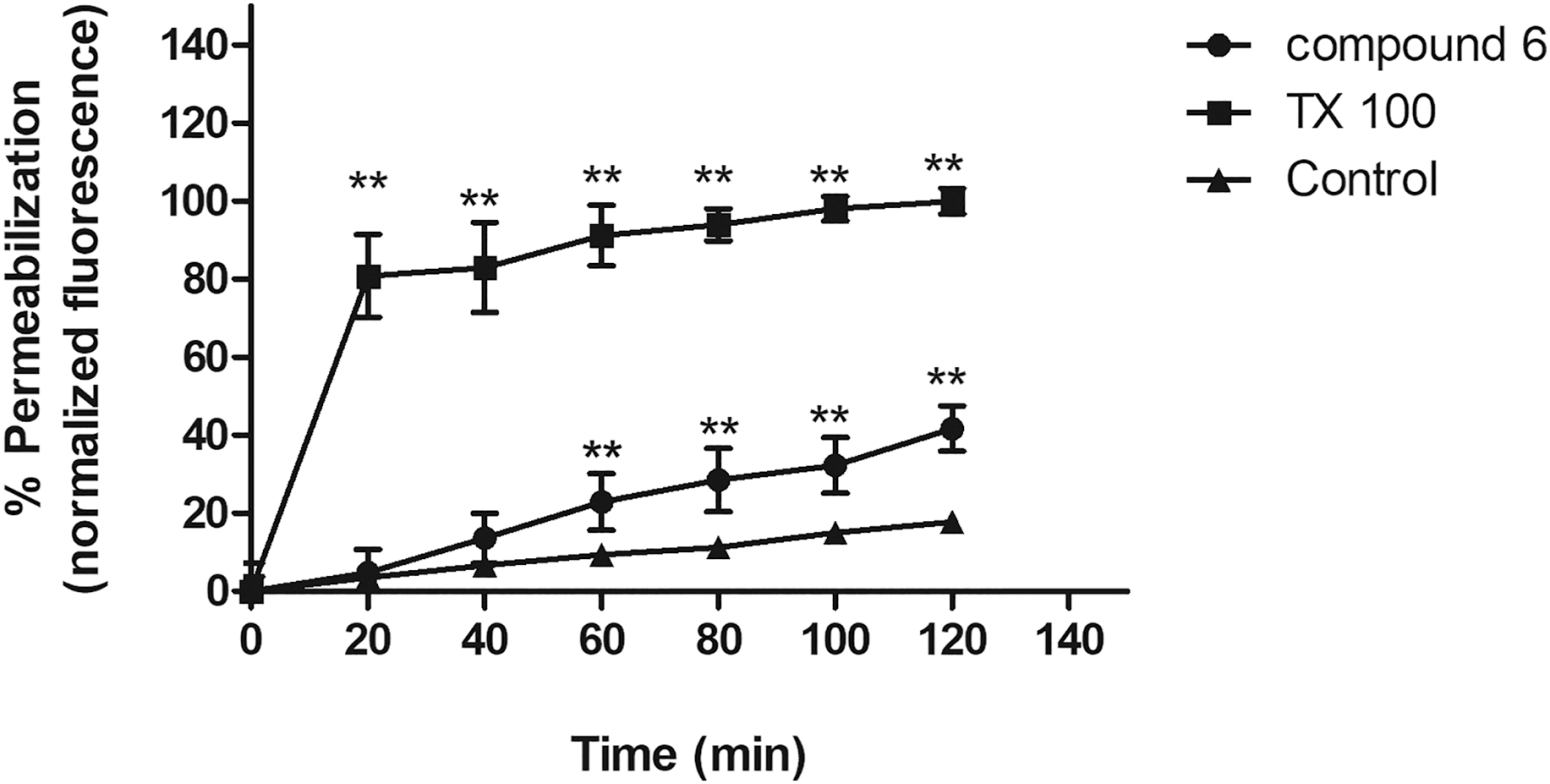
Evaluation of plasma membrane permeability. *T. cruzi* trypomastigotes were treated with Cytosporone B (compound **6**) (55 μM), TX-100 (0.5%), and untreated parasites were investigated by SYTOX Green. The permeabilization was monitored spectrofluorimetrically every 20 min. A representative experiment is shown. **p < 0.05.

**Figure 3:**
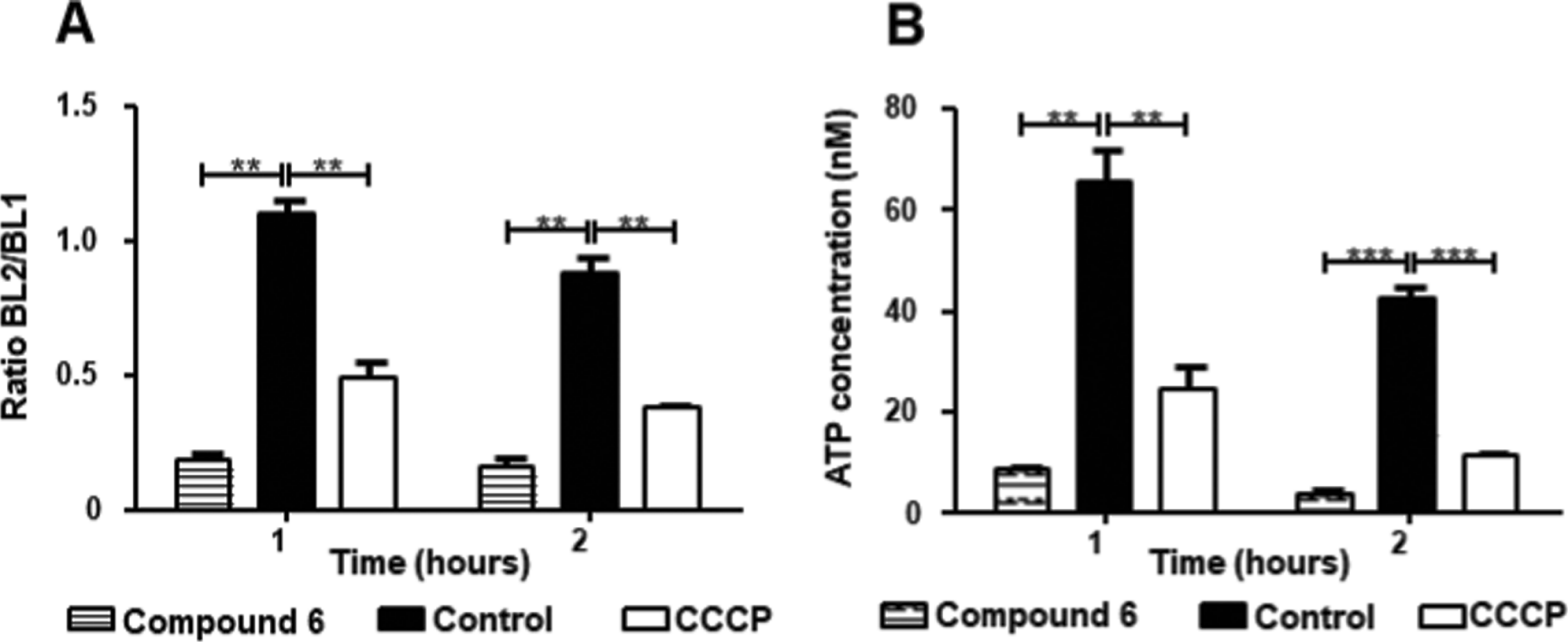
Evaluation of mitochondrial membrane electric potential (ΔΨ_m_) and ATP levels. *T. cruzi* trypomastigotes were incubated for 1 or 2 h with Cytosporone B (**6**) (55 μM). (A) ΔΨ_m_ was investigated with JC-1 dye by flow cytometry and reported as the ratio between the emission channels BL2/BL1. (B) ATP concentration was measured by luciferin/luciferase luminescent kit in a plate reader luminometer. In both analyses, untreated and treated promastigotes with CCCP were used to achieve minimal and maximal depolarization, respectively. A representative experiment is shown. ***p < 0.005 and **p < 0.05.

## Results and Discussion

Few endophytic fungi from *C. sylvestris* have been investigated for metabolites, including alkaloids and polyketide derivatives from *Colletotrichum crassipes*, as well as diketopiperazines and simple phenolics from *Xylaria* sp.^16^ It has been found that *Phomopsis* sp. from *C. arborea* can produce cytosporones and dothiorelones.^8^ Various compounds have been identified in *Phomopsis* spp., including cyclopeptides, cytochalasins, dibenzofuranodione, isocoumarins, macrolactones, naphthalenes, and xanthones. These compounds have potential applications, including medicine and agriculture. It is important to continue exploring and utilizing these natural products in a sustainable manner.^17^ The mycobiota of *Casearia* is poorly known and understood, and *Casearia* plants may be a promising source for discovering endophytic fungi that could produce more complex and diverse natural products for bioactivity’s prospection.

In the present work, the activity of six cytosporone compounds (**1**–**6**) and cytochalasin H (**7**) from endophytic fungi, isolated from leaves of *C. arborea*, was tested against *T. cruzi*, the protozoan agent of Chagas disease. Dothiorelone, cytosporone, and cytochalasin are secondary metabolites found in endophytic fungi with potential antibacterial activity, cancer, and antiparasitic properties.^18,19^ In this work, the anti-*T. cruzi* potential of the compounds **1–7** was evaluated against trypomastigotes and intracellular amastigotes to determine the IC_50_ values (Table 1).

It is important to note the distinction in the maximum concentration tested between the two assays. While extracellular trypomastigotes were assayed up to 150 μM against trypomastigotes, the evaluation against intracellular amastigotes was performed at a maximum of 60 μM. This lower limit was intentionally implemented to mitigate potential toxicity to the host cells, ensuring that any observed effect in parasite was genuinely attributed to the compound’s effect rather than secondary host cell damage. Furthermore, performing the concentration at 60 μM allows us to comply with established Drugs for Neglected Diseases initiative (DND*i*) criteria for prioritizing novel hit compounds, streamlining the initial screening results for future hit-to-lead optimization efforts.

The compounds **1–3** (Dothiorelone A, B, and P) showed no activity against trypomastigotes and intracellular amastigotes to the maximum tested concentration (150 μM). Compounds **4** (Dothiorelone Q) and **5** (Cytosporone A) were active against intracellular amastigotes with IC_50_ values of 43 μM and 29.9 μM, respectively. Furthermore, **7** (Cytochalasin H) was active only in trypomastigotes with an IC_50_ of 47.9 μM, while **6** (Cytosporone B) demonstrated activities against both parasite forms with IC_50_ values of 36.5 μM against trypomastigotes and 9.1 μM against amastigotes. The cytotoxicity values against NCTC cells were also investigated, and the compounds showed no relevant toxicity for the mammalian cells at the highest tested concentration (200 μM). The selectivity index (Supporting Information), given by the relationship between the mammalian cytotoxicity in NCTC cells and the activity against intracellular amastigotes, was calculated and resulted in Supporting Information > 4.6 for Dothiorelone Q (**4**), Supporting Information > 6.7 for compound Cytosporone A (**5**), and Supporting Information > 22 for Cytosporone B (**6**).

In the literature, some promising biological activities have been described for compounds **4**, **5,** and **6**.^20^ Compound **4** played a role in the inhibition of the TFG-β induced by the activation of human lung fibroblast MRC-5 cells.^18^ Compound **5** presented an important role in inhibition for germination and growth of plants, and **6** showed potent antibacterial activity; both compounds also demonstrated antimicrobial activity against *Staphylococcus aureus*, *Enterococcus faecalis*, *Escherichia coli*, *Candida albicans*.^21–24^ Csn-B (**6**) also showed an important role as an agonist of Nur77, a nuclear receptor involved in several biological processes such as cell proliferation, differentiation, apoptosis, metabolism, and development.^25^ It is important to highlight that this work reports for the first time the activity of **4**, **5**, and **6** against protozoan parasites.

Csn-B (**6**) (Figure 1) was designated the most selective agent of the panel, exhibiting potent activity against both relevant stages of *T. cruzi*. Specifically, it achieved excellent IC_50_ values for a hit compound against trypomastigotes and intracellular amastigotes. It also demonstrated important characteristics of hit compounds, defined by the DND*i* (Drugs for Neglected Diseases initiative) criteria for new hits. In addition, Csn-B meets most of these criteria, including IC_50_ < 10 μM and Supporting Information > 10, and could eventually be addressed to a hit-to-lead optimization phase.^26^ Given its superior selectivity index, a key criterion for drug discovery progression, Csn-B (**6**) was prioritized for mechanistic investigation. Subsequent studies focused on understanding its lethal action profile using advanced spectrofluorimetric and flow cytometry assays.

The plasma membrane is the main structure for the maintenance of cell morphology and physiology. In unicellular protozoan parasites such as *T. cruzi*, this organelle is important to homeostasis maintenance and interaction with the host cell during cell invasion. SYTOX Green is a fluorescent probe that was used to investigate alterations in the plasma membrane of trypomastigotes in the presence of compound Csn-B. According to the data presented in Figure 2, no alteration in the plasma membrane was observed when compared to untreated parasites.^27^

Trypanosomatids present one single mitochondrion that fills up to 12% of the cell volume and is essential for the parasite’s survival, and this organelle has become a drug target. In this context, the stability of the mitochondrial membrane potential (ΔΨ_m_) and the ATP levels are needed for the cell function.^28,29^

The ΔΨ_m_ studies used JC-1 dye as a probe, a cationic carbocyanine compound.^30^ Trypomastigotes were incubated for 1 and 2 h in the presence of Csn-B and after the treatment, a significant depolarization of the mitochondria was observed (Figure 3A). In the literature, studies demonstrated that *T. cruzi* treated with benznidazole presented a respiratory chain inhibition and depolarization of the mitochondrial membrane potential.^31^ Csn-B induced similar effects, suggesting a possible inhibition of the respiratory chain.

Perturbation in mitochondrial membrane potential promotes alterations in NADH/NAD+, ATP/ADP, and can reduce ATP levels.^32^ The results showed that Csn-B promoted a significant decrease in ATP levels after 1 and 2 h of treatment. Since mitochondria is the organelle responsible for ATP production for several cellular activities, alterations in this organelle can promote changes in the ATP levels, disturbing the cell homeostasis.^32,33^ Thus, this ATP reduction induced by Csn-B may be attributed to the observed mitochondrial damage.

Furthermore, our findings indicate that Csn-B (compound 6) exerts its trypanocidal effect through a mechanism distinct from that of current standard drug benznidazole. This compound requires enzymatic activation by parasite nitroreductases, leading to the formation of reactive intermediates that induce oxidative stress and covalent modification of DNA and proteins. In contrast, Csn-B acts primarily by disrupting mitochondrial homeostasis, as evidenced by the depolarization of the mitochondrial membrane potential and a significant reduction in intracellular ATP levels. These effects point to a direct impairment of mitochondrial function and energy metabolism rather than generalized oxidative stress. The novelty and biological relevance of Csn-B lie in its targeting of the parasite’s single mitochondrion, a unique and essential organelle in *T. cruzi*, making it particularly vulnerable to perturbations in mitochondrial bioenergetics. Mitochondrial dysfunction is known to trigger a cascade of lethal events in kinetoplastids, including loss of redox balance, impairment of ATP synthesis, and collapse of essential metabolic pathways. Therefore, Csn-B represents a mechanistically distinct and potentially complementary approach to trypanocidal therapy. This mitochondrial mode of action opens new perspectives for drug discovery in *T. cruzi*, emphasizing the potential of synergistic agents to current nitro-based chemotherapies.

Further, suggesting a structure-activity relationship for the active compounds 4–6, Csn-B was observed to exhibit higher selectivity (IS 22.0), likely due to its lipophilic (hydrophobic) hydrocarbon side chain, which is also found in Csn-A (IS 6.7). This lipophilic chain may interact with mitochondrial membrane lipids, facilitating depolarization, especially since Csn-B contains an ester group—absent in Csn-A (IS 6.7) and Dot-Q (IS 4.6). The presence of a carboxylic acid group decreases the depolarizing activity of Csn-A and Dot-Q. An aqueous medium with a pH ∼8 (due to the Hanks’ Balanced Salt solution) will shift the equilibrium toward the conjugate base (carboxylate) form, making Dot-Q and Csn-A carry a greater negative charge. Therefore, Dot-Q and Csn-A have a higher formal charge than the ester group of Csn-B, which may reduce their effect on membrane depolarization. As a result, the lipophilic side chain and the ester group can more effectively cause mitochondrial membrane depolarization. When the side chain is oxidized and polarized, its lipophilicity decreases, reducing its effectiveness, as seen with Dot-P. In other words, the presence of an OH group attached to C14 decreases the activity, and if OH is present at C15, there will be no activity, even if the ester group remains (as seen in Dot-A and Dot-B).

Finally, when comparing the structure of Csn-B with the control, benznidazole, a structural similarity between the two is observed. The ester group is slightly polarized, as is the nitro group. Both have a central aromatic ring—phenol in Csn-B and imidazole in benznidazole. Additionally, the side chain in both compounds is lipophilic, allowing interaction with the membrane (Suppl. D).

## Conclusions

In this work, seven compounds were isolated from endophytic fungi associated with *C. arborea* leaves and were tested against *T. cruzi*. Cytosporone B (compound **6**) exhibits the most promising activity against intracellular and extracellular forms, and the mechanism of action was investigated. This compound induced mitochondrial failures, causing the depolarization of the membrane, leading to a bioenergetic breakdown and consequent ATP decrease. This targeting of the kinetoplastid mitochondrion is a key finding, suggesting Cytosporone B as a novel hit compound and a starting point for the development of new antitrypanosomal agents. Moving forward, these results contribute to inform the next stage of hit-to-lead optimization, necessitating comprehensive structure-activity relationship (SAR) studies around the Csn-B scaffold to refine its potency, enhance its selectivity index, and optimize its pharmacokinetic properties, thereby positioning it as a promising prototype for future exploration against Chagas disease.

## Supplementary Material



## Notes

All structures of compounds 1–7, spectroscopic, spectrometric, and chromatography conditions, including Classical Molecular Networking workflow parameters, were organized in the file named freely available (PDF)

## Abbreviations

ATP: (adenosine triphosphate)
CC_50_: (50% cytotoxic concentration)
CO_2_: (carbon dioxide)
Csn-B: (Cytosporone B)
DNDi: (Drugs for Neglected Disease initiative)
IC_50_: (50% inhibitory concentration)
HBSS: (HANKS balanced salt solution)
HPLC-DAD: (high performance liquid chromatography coupled with diode array detector)
HRMS/MS: (high-resolution *tandem* mass spectrometry)
MTT: ((3-(4,5-dimethylthiazol-2-yl)-2,5-diphenyltetrazolium bromide)
NMR: (nuclear magnetic resonance)
ΔΨm: (mitochondrial membrane electric potential).
