# Supplementary material for: Cytosporone B Affects ATP Production of Trypanosoma cruzi: A Lethal Action Investigation
Source: ACS Omega. 2025 Dec 10;10(50):62060–6. doi: 10.1021/acsomega.5c09059 (PMC12750222; doi:10.1021/acsomega.5c09059)

SUPPLEMENTARY MATERIALS

Cytosporone B affects ATP production of *Trypanosoma cruzi*: A Lethal Action Investigation

Augusto Leonardo dos Santos1†, Maiara Amaral2†, Erica V. de Castro Levatti2, Maiara Romanelli2, Marcelo J. Pena Ferreira3, Andre G. Tempone2*, Patrícia Sartorelli1*

1Institute of Environmental, Chemical and Pharmaceutical Sciences, Department of Chemistry, Federal University of São Paulo, 09972-270, São Paulo, Brazil.
2Laboratory of Pathophysiology, Instituto Butantan, 05503-900, São Paulo, Brazil.
3Botany Department, Institute of Biosciences, University of São Paulo, São Paulo, 05508-090, Brazil.
†These authors contributed equally to this work.

List of Supplementary Materials

A: Structures of compounds 1-7. Compound 1 (Dothiorelone A), 2 (Dothiorelone B), 3 (Dothiorelone P), 4 (Dothiorelone Q), 5 (Cytosporone A), 6 (Cytosporone B), and 7 (Cytochalasin H), **page 2.**

B: Classical Molecular Network worflow parameters, **page 3**.

C: Spectroscopy, spectrometric parameters and chromatographic conditions to isolate and purify, ando to identify compounds 1-7, **page 4**.

D: Structure-Activity Relationship for compounds 4-6 and Benznidazole, **page 5**.

Suppl. Mat. A: Structures of compounds 1-7. Compound 1 (Dothiorelone A), 2 (Dothiorelone B), 3 (Dothiorelone P), 4 (Dothiorelone Q), 5 (Cytosporone A), 6 (Cytosporone B), and 7 (Cytochalasin H).

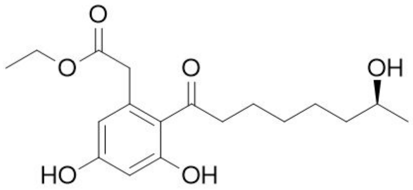

Compound 1
Dothiorelone A

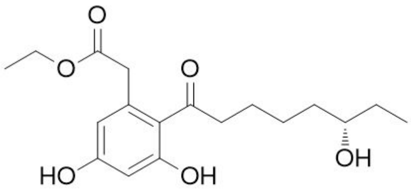

Compound 2
Dothiorelone B

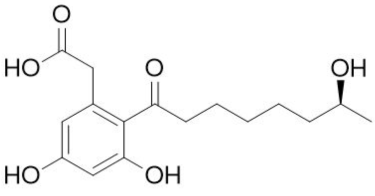

Compound 3
Dothiorelone P

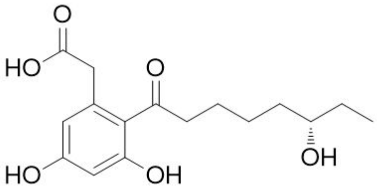

Compound 4
Dothiorelone Q

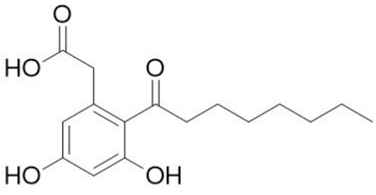

Compound 5
Cytosporone A

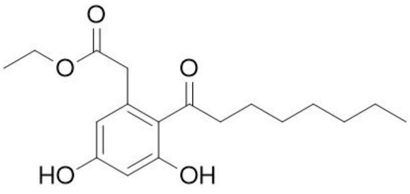

Compound 6
Cytosporone B

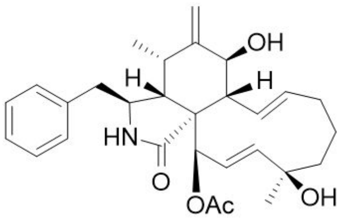

Compound 7
Cytochalasin H

References: https://doi.org/10.1016/j.phytol.2020.11.020; https://doi.org/10.3390/metabo12100903;

Suppl. Mat. B: Classical Molecular Network worflow parameters.

For compounds 1 – 6 (https://doi.org/10.1016/j.phytol.2020.11.020)

All data obtained from Bruker micrOTOF-QII-ESI were converted to the ".mzML" extension, to perform the dereplication on GNPS for database matches, using MSConvert (Kessner et al., 2008) and conferred in SeeMS, both are free softwares from Proteowizard®. The ".mzML" archives were uploaded to the Global Natural Products Social Molecular Networking Web server using WinSCP to create the molecular networking (Wang et al., 2016). The data were treated with in GNPS Data Analysis platform removing fragments of ±17 Da of precursor m/z. MS/MS spectra were filtered choosing only the six top fragments in the ±50 Da window in all range of spectra. In the basic options the tolerance ions were set to 0.03 Da and for precursor ions and 0.05 Da for MS/MS fragment ions. A network was than created using the MS-Cluster with clusters containing at least two spectra (Frank et al., 2008), according to cosine score above 0.75 and more than 6 matched peaks. Further, edges between two nodes were kept in the network if and only if each of the nodes appeared in each other's respective top ten most similar nodes. The maximum size of a molecular family was set to 100, and the lowest scoring edges were removed from molecular families until the molecular family size was below this threshold. The spectra in the network were then searched against GNPS' spectral libraries. The library spectra were filtered in the same manner as the input data. All matches kept between network spectra and library spectra were required to have a score above 0.60 and at least 6 matched peaks.

For compound 7 (https://doi.org/10.3390/metabo12100903)

All data obtained from Bruker micrOTOF-QII were converted to the ".mzML" extension, to perform the dereplication on GNPS for database matches, using MSConvert and conferred in SeeMS, both are free software from Proteowizard®. The ".mzML" archives were uploaded to the Global Natural Products Social Molecular Network Web server using WinSCP to create the molecular network [26]. The data were treated within the GNPS Data Analysis platform removing fragments of ±17 Da of precursor m/z. HRMS-MS spectra were filtered choosing only the six top fragments in the ±50 Da window in all ranges of spectra. In the basic options, the mass tolerance for precursor ions and fragment ions was set to 0.02 Da. A network was created using the MS-Cluster with a minimum cluster size containing two spectra according to a cosine score above 0.7 and more than three matched peaks. Further, edges between two nodes were kept in the network if, and only if, each of the nodes appeared in each other's respective top 10 most similar nodes. The maximum size of a molecular family was set to 100, and the lowest scoring edges were removed from molecular families until the molecular family size was below this threshold. The spectra in the network were then searched against GNPS' spectral libraries. The library spectra were filtered in the same manner as the input data. All matches kept between network spectra and library spectra were required to have a score threshold above 0.60 and at least four matched peaks (MSV000086335, doi:10.25345/C5CJ5B). Clusters detected in blank were removed from the networking that includes spectral data from solvents used in the extraction and chromatographic procedures (dried hexane, chloroform, ethyl acetate, methanol). The molecular networking view and edition were performed in Cytoscape v.3.8.2.

Suppl. Mat. C: Spectroscopy, spectrometric parameters and chromatographic conditions to isolate and purify, ando to identify compounds 1-7.

For compounds 1 – 6 (https://doi.org/10.1016/j.phytol.2020.11.020)

1H and 13C NMR (1D and 2D) spectra were recorded at 500.13 and 125.77 MHz, respectively, in the Ultrashield 500 spectrometer (BrukerBiospin, Germany). The instrument was equipped with 5-mm trinuclear, inverse detection probe with z-gradient (TXI). The temperature was controlled by a BCU I accessory at 25 ◦C. Data acquired on 300.13 MHz (1H) and at 75.77 MHz (13C) in Ultrashield 300 Avance III spectrometer (Bruker-Biospin, Germany). Methanol-d4 (Sigma-Aldrich) was used as the solvent and internal standard.

UHPLC-HR-ESI-MS/MS data was obtained on Shimadzu Nexera X2 system (Shimadzu, Japan) equipped with a SPD-M20A Proeminence Diode Array detector and coupled to a quadrupole time-of-flight mass spectrometer (MicroTOF-QII; Bruker Daltonics, USA) equipped with ESI operating in positive ion mode at 18,000 FWHM of mass resolution.

For compound chromatography analyzes, a Kinetex columm C18 (Core-Shell – 2.6 µm - 100.0 mm × 2.1 mm) was applied. All solvents were AR grade. The HPLC-DAD analysis was performed in the Ultimate 3000 liquid chromatograph with UV-DAD detector (Dionex) using a Luna RP-18 column (5 µm, 150 × 5 mm) (Phenomenex). A silica gel (Merck, 230–400 mesh) and Sephadex LH-20 (Sigma-Aldrich) were used for the CC separation, whereas the silica gel 60 PF254 plates (Merck) was used for the analytical TLC and the spots were visualized under UV light (254 nm).

The specific optical rotation $[\alpha]^{D}_{25}$ of compounds 2 and 3 (c 0.1) was measured in a digital polarimeter Krüss (P3000) using MeOH as stan dard solvent at room temperature.

For compound 7 (https://doi.org/10.3390/metabo12100903)

The extract obtained from Diaporthe sp. CarGL8 incubation in rice furnished a brown and amorphous extract in ethyl acetate which presented cytotoxic effects against three tumor cell lines. The crude extract was partitioned with hexane and MeOH:H2O (9:1). The hydromethanolic phase (400 mg) was then subjected to a chromatographic column (CC) over SiO2 using a gradient in CHCl3:MeOH starting at 95:5, increasing the polarity and ending in 100% MeOH furnishing five group fractions (I–V). Group II was selected by the LC-DAD profile for further purification due to the presence of a major compound. Fraction II was subjected to Sephadex LH-20 CC using methanol as the mobile phase furnishing five fractions (II-1 to II-5). The LC-DAD revealed one chromatographic band in group II-1 (90 mg) at 220 nm with a low absorption chromophore UVmax 254 nm. The compound II-1 was then analyzed by NMR (same as used for compounds 1-6) and HRMS-MS for structural identification.

Liquid chromatography coupled to high-resolution tandem mass spectra (LC-HRMS/MS) data were acquired on Shimadzu Nexera X2 ultra-performance liquid chromatography system (Shimadzu, Japan) equipped with an SPD-M20A Proeminence Diode Array detector, using a reverse phase Kinetex EVO C18 column (2.6 µm—100.0 mm × 2.1 mm). All solvents were spectroscopic grade. The LC system was coupled to a QTOF mass spectrometer equipped with an electrospray (ESI) operating in positive ion mode at 18,000 FWHM of mass resolution (MicrOTOF-QII; Bruker Daltonics, Billerica, MA, USA). Dried EtOAc crude extracts (5 mg) were dissolved in 1 mL of MeOH and centrifuged at 15,000 rpm for 10 min, 20 ◦C. The supernatant (500 µL) was transferred to a vial and the same volume of MeOH:H2O (1:1) was added. Compounds (1 mg mL−1) previously obtained

from Phomopsis sp. CarGL23 [21] were used to guide the cytosporone and dothiorelone derivatives annotation in the molecular network. Samples were injected (3 μL) into the LC system at 50 ◦C in a column chamber. The chromatographic separation was performed using the mobile phase in a gradient of A (H2O + 0.1% formic acid) and B (ACN + 0.1% formic acid), and a flow of 350 μL min−1 for the following method: 0–2 min 5% B, 2–13 min 5% to 98% B, 13–16 min 98% B, 16–18 min 98% to 5% B, 18–21 min 5% B for the column stabilization for the next injection. HRMS-MS data were obtained from a quadrupole tandem time-of-flight (QTOF) mass analyzer under positive mode ESI at a mass range of m/z 50–1200. The positive ionization on ESI was set as follows: capillarity voltage of 4500 V and end plate offset at 500 V, dry gas (N2) at a flow of 8.0 mL min−1, a pressure of 4.0 Bar, and temperature of 200 ◦C. The collision-induced dissociation (CID) energy was set at 25 eV and auto-MS-MS were performed for three precursor ions, with active exclusion after three spectra and release after 1 min, reconsidering the precursor if the current intensity is five times more intense than the previous intensity. Calibration was set at less than 2 ppm using sodium formate.

D: Structure-Activity Relationship for compounds 4-6 and Benznidazole.

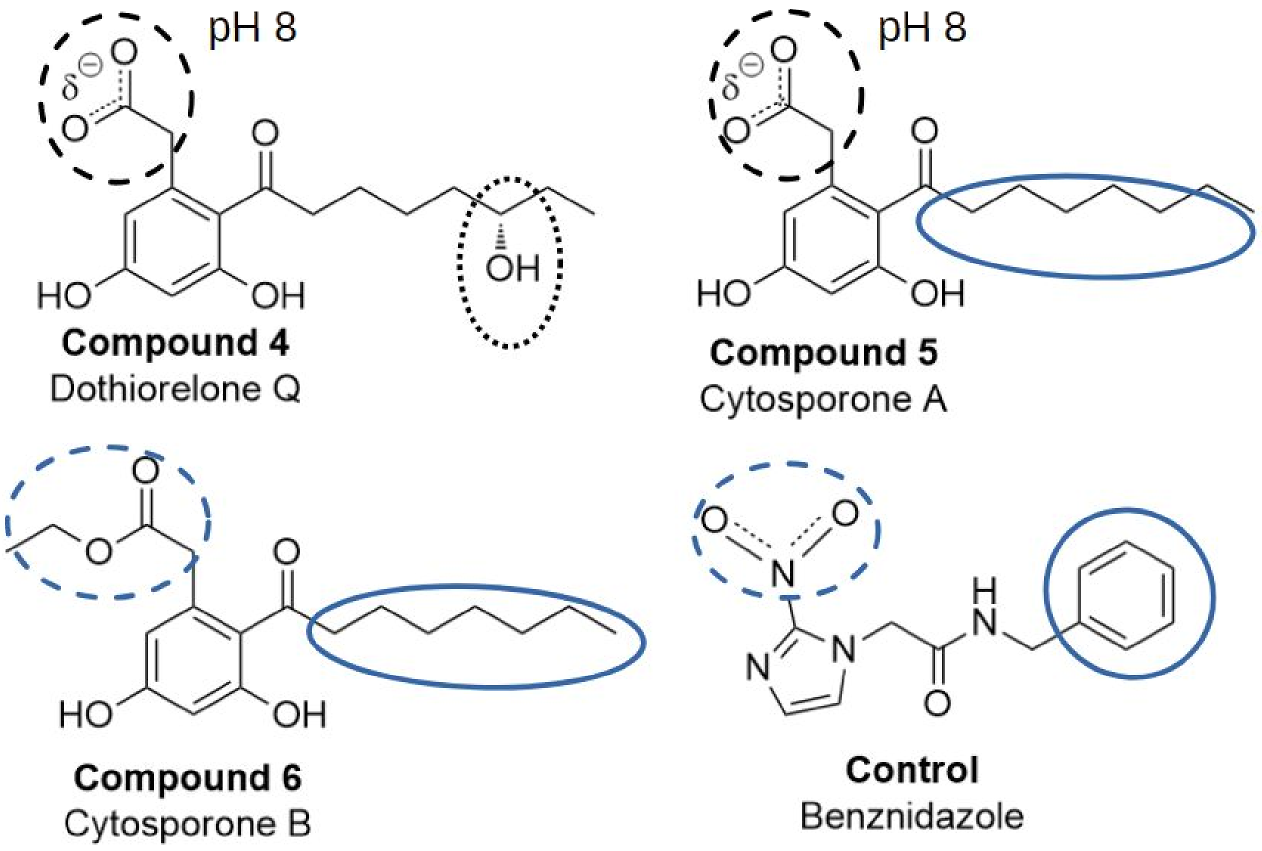

Supplement: Supplementary file 1 [file ao5c09059_si_001.pdf]
